# Assessing Worry About Affording Healthcare in a General Population Sample

**DOI:** 10.3389/fpsyg.2019.02622

**Published:** 2019-11-22

**Authors:** Salene M. W. Jones, Yuxian Du, Laura Panattoni, Nora B. Henrikson

**Affiliations:** ^1^Fred Hutchinson Cancer Research Center, Seattle, WA, United States; ^2^Bayer Healthcare U.S. LLC, Whippany, NJ, United States; ^3^Kaiser Permanente Washington Health Research Institute, Seattle, WA, United States

**Keywords:** financial anxiety, economic well-being, financial toxicity, economic problems, financial burden

## Abstract

This study adapted a measure on worry about affording healthcare. The financial costs of healthcare are increasingly being shifted to patients. Financial burden from healthcare costs can be material (such as bankruptcy) or psychological. Psychological distress can be either worry about affording future care or distress due to material consequences and, despite evidence from clinical psychology that differentiates these types of emotional symptoms, this distinction has largely been ignored for financial burden in healthcare. We adapted a worry about affording healthcare scale for use in the general population (*n* = 398) to facilitate comparisons between disease groups and across countries. Participants completed a survey through an online platform. The worry about affording healthcare measure showed good reliability and validity through associations with quality of life (QOL) and measures of other types of financial burden. Worry about affording healthcare was also associated with cost-related non-adherence to medical care. Future research on patient QOL should consider worry about affording healthcare.

## Introduction

Healthcare costs have been rising (Gordon N. et al., 2017). Theories and systematic reviews of financial burden in healthcare have distinguished material financial burden (bankruptcy, draining savings) due to healthcare costs from psychological financial burden (Altice et al., 2017; Gordon L.G. et al., 2017). Material financial burden is also called financial strain or objective financial burden due to the focus on tangible stressors such as not having enough money for medications. Psychological financial burden is also called subjective financial burden or financial stress due to the focus on emotions and perceptions related to financial health.

Psychological financial burden includes two distinct concepts: (1) the distress and depressive-type symptoms (such as feeling down or having little interest in activities) that result from the stress of material financial burden, and (2) worry and anxiety about affording future healthcare (Beck and Haigh, 2014). This distinction is particularly important as research has shown anxiety and depression are associated with different outcomes: depression and rumination is associated with insomnia, while anxiety and worry is associated with cortisol and heart rate variability (Carney et al., 2010; Ciesla et al., 2011; Aldao et al., 2013; Crowley et al., 2015; Lewis et al., 2017). Anxiety and worry need to reference a specific topic, rather than be general, to predict quality of life (QOL) and other outcomes (Jerndal et al., 2010; Buss et al., 2011; Reck et al., 2013). Previous work on financial burden has largely ignored the difference between different types of psychological financial burden (distress vs. general anxiety vs. worry specific to affording healthcare). Measures distinguishing worry about affording healthcare from other types of psychological financial burden, including general financial worry and anxiety, are needed.

The theory of conservation of resources (Hobfoll, 1989) is particularly useful for understanding the role of worry about affording healthcare. In the conservation of resources theory, people are motivated to prevent loss of resources such as money, and the threat of losing resources may be more motivating than actual loss (Halbesleben et al., 2014). For this reason, we elected to focus on worry rather than anxiety as it might better assess the perceived threat of resource loss, since worry is more cognitive while anxiety reflects emotions. Worry about affording healthcare would then represent a concern about a loss of resources that motivates cost-related non-adherence to medical treatments as a way to conserve resources. This could be motivating even for people without medical conditions as it could lead to less use of preventive care like cancer screenings, dental visits and optometry services.

To address the lack of a measure focusing on worry about affording healthcare measure, this study adapted the worry about affording healthcare scale (WAHS) for use in the general population. The measure was initially developed in Multiple Sclerosis patients living in the United States (Jones and Amtmann, 2014). The measure was specifically adapted to facilitate comparisons across countries and disease groups as well as comparisons between disease groups and healthy controls. Even in countries with nationalized healthcare, costs are increasingly being shifted to the patient (Hossein and Gerard, 2013). Countries with universal healthcare, such as the United Kingdom, Canada, and Australia, still have supplemental private insurance (International Health Care System Profiles, 2019) suggesting concerns about cost may still be an issue. To facilitate comparisons of how worry about affording healthcare may differentially affect QOL and healthcare use between countries and groups, we created a measure of worry about affording healthcare that could be used in nearly any setting or disease group.

## Methods

### Sample and Procedures

Our sample consisted of 398 adults recruited through Prolific Academic, a crowdsourcing website that helps people participate in online studies that has been used in numerous peer-reviewed publications (Palan and Schitter, 2018). Previous research has shown that the Prolific platform is likely less biased than alternatives such as in-person survey panels of students and Mechanical Turk (Peer et al., 2017). This is possibly because Prolific was originally created to support academic research whereas other platforms were created for private industry. Once a survey is posted to Prolific Academic, eligible participants either receive notification that a new study has been posted or they regularly check the website for new studies. Potential participants then click the survey link, read the consent form and, if they agree to the study, complete the survey. Participants completing the survey received USD$8. Participants had to be 18 years of age and able to read and write English. Participants also had to answer three of four attention check questions correctly. The survey was administered through Limesurvey on September 4, 2018. Informed consent was obtained through the online survey. We sought feedback from a paid patient consultant about changes to the worry about affording healthcare measure that would make the measure applicable outside the United States. Based on her feedback, we elected to test two versions of the wording for the WAHS. Limesurvey randomly assigned participants to receive one of the two versions of the survey wording: concern about affording healthcare or fearful about affording healthcare. This study was reviewed by the Fred Hutchinson Cancer Research Center institutional review board. The datasets for this manuscript are not publicly available because information on medical conditions was collected. Requests to access the datasets should be directed to the corresponding author.

### Measures

#### Worry About Affording Healthcare Scale (WAHS)

The WAHS was originally created as a five-item scale to assess worry about affording healthcare in Multiple Sclerosis patients in the United States (Jones and Amtmann, 2014). However, due to substantial differences in the United States-based healthcare system compared to other countries, we edited the questions and had a patient consultant review the revised items. We edited the items so they would be applicable outside the United States and for other disease groups. We made the following changes for the general population version, we removed two questions about affording premiums or having insurance canceled. We changed one item on income going down and not being able to afford premiums, so it instead asked about income going down and not being able to afford healthcare services. We also added an item on worry about affording medical devices. Two questions on worry about affording healthcare services and prescriptions remained unchanged. The final scale had four items: income going down; affording healthcare services; affording prescriptions; and affording medical devices (Supplementary Materials). Each item is rated on a four-point scale (0 = “not at all concerned/fearful”, 1 = “not too concerned/fearful”, 2 = “Somewhat concerned/fearful,” and 3 = “Very concerned/fearful”). The total score is calculated by summing the items and ranges from 0 to 12.

#### General Financial Health

Participants completed two measures on general financial health for validation of the WAHS. First, the Financial Anxiety Scale (FAS) is a seven-item measure with items corresponding to the symptoms of Generalized Anxiety Disorder but specific to financial problems (American Psychiatric Association [APA], 2000) and items are rated on a 1–7 scale (Archuleta et al., 2013). The FAS has been shown to have good reliability and validity (Archuleta et al., 2013). Second, the Financial Well-being Scale (FWS) from the Consumer Financial Protection Bureau (CFPB) was administered. The FWS has ten items, each rated on a five-point scale, and five items are reverse-scored (Consumer Financial Protection Bureau, 2015). Raw sum scores are then converted to an item response theory (IRT) based scoring using a conversion table. For both the FAS and FWS, higher scores indicate better financial health.

#### General Anxiety

Also, for validation of the WAHS, participants completed the four-item version of the Patient-Reported Outcomes Measurement Information System (PROMIS) Anxiety Scale (Cella et al., 2010). Participants rate four symptoms of anxiety on a five-point scale. Raw sum scores are also converted to IRT-based *t*-scores (population mean of 50, standard deviation of 10) using a conversion table. Higher scores indicate more anxiety.

#### Quality of Life (QOL)

We assessed QOL using the two-item PROMIS measure for mental health (Hays et al., 2017). Each item is rated on a five-point scale. Raw scores are also converted to IRT-based *t*-scores. Higher scores indicate better QOL.

#### Medical Conditions and Demographics

Participants also reported their age, gender, education, marital status, region of current residence, and race/ethnicity. Participants also completed the Self-administered Comorbidity Questionnaire (SCQ) (Sangha et al., 2003). The SCQ has participants self-report diagnoses with 12 health conditions, whether they receive treatment and whether the condition limits their activities. Scores range from 0 to 36 on the SCQ with higher scores indicating more medical burden. The 12 medical conditions on the SCQ are: heart disease, high blood pressure (hypertension), lung disease, diabetes, ulcers or stomach disease, kidney disease, liver disease, anemia or blood disease, depression, osteoarthritis, and back pain or rheumatoid arthritis.

#### Cost-Related Non-adherence to Medical Care

Participants were also asked to respond to five questions about not adhering to medical recommendations due to costs. Items included in the measure were cutting back on prescriptions, not buying prescriptions, not making doctors’ appointments, not using medical services, and not having medical tests. Each item was rated on a three-point scale (never, sometimes, and often). If participants mark “sometimes” or “often” for any item, they are classified as experiencing cost-related non-adherence. This measure has previously been shown to have validity and reliability in those with cancer (Khera et al., 2014).

### Psychometric Analyses

Reliability of the two versions of the WAHS was assessed using Cronbach’s alpha. We assessed validity of the two versions of the WAHS through Spearman’s correlations with the FAS, FWS, PROMIS-Anxiety Scale, PROMIS QOL Scales, and the SCQ. We also conducted two multiple linear regressions with the scores from the WAHS as criterion variables. The predictor variables were age, gender (male vs. female and non-binary), race/ethnicity (monoracial, non-Hispanic white vs. other groups), education (graduate degree vs. bachelor’s degree vs. some college, associate’s or technical degree vs. high school diploma or less), marital status (single vs. long-term vs. married), region (United States vs. Europe vs. other regions), and cost-related non-adherence. Gender, race/ethnicity, education, marital status, and region used dummy coded variables. We also compared the means between the two different wordings of the WAHS using a *t*-test, and we compared the specific items between the versions of the WAHS using differential item function (DIF). DIF uses IRT to determine whether the wording biased responses to specific items while controlling for the overall level of worry about affording healthcare. IRT creates a logistic model for each item using two parameter sets per item (Samejima, 1969). The first parameter estimates how accurately the item reflects the underlying construct, worry about affording healthcare in this case. The second parameter set estimates how much of the construct a person needs (or how worried someone needs to be) before they mark a particular response category for that item and are often called ability parameters. In DIF, separate IRT models are created for two groups (wording of the WAHS in this case) and compared for statistically significant differences in the accuracy and ability parameters while controlling for overall level of the construct. A lack of DIF would ensure that any differences seen between the two versions of the WAHS was due to the wording change (concerned vs. fearful) and not due to an interaction of a specific item with the wording. We also conducted a receiver operating curve (ROC) analysis for the WAHS with cost-related non-adherence as the outcome. IRT analyses were conducted using IRTPRO 4.2. All other analyses were conducted using SPSS 25 or 26.

## Results

### Sample Description

We originally recruited 400 participants but excluded two because they did not correctly answer three out of the four attention check questions. All other 398 participants correctly answered three or four of the four attention check questions. Descriptive statistics for the sample are reported in Table 1. Two-thirds of the sample (66.8%, *n* = 266) reported at least one of the 12 medical conditions on the survey. The mean score on the SCQ was 2.63 (SD = 2.88) with scores ranging from 0 to 18. The most common conditions were depression (44.7%, *n* = 178), back pain (38.4%, *n* = 153), high blood pressure (11.3%, *n* = 45), anemia (7.5%, *n* = 30), and osteoarthritis (7.5%, *n* = 30). In United States Dollars, the mean yearly income was $31,689 and the median was $23,377. Consistent with previous studies using Prolific Academic, participants were young and had some post high school education. The mean for the WAHS-concerned was 4.05 (SD = 3.74), and the WAHS-fearful was 4.04 (SD = 3.92); these were not significantly different (*t*(396) = 0.034, *p* = 0.973).

**TABLE 1:** Sample description (*n* = 398) and standardized regression coefficients with WAHS from multiple linear regressions.

		**Standardized regression coefficients**	
		
**Characteristic**	***N* (%) or mean (*SD*)**	**Outcome: WAHS-concerned (*n* = 201)**	**Outcome: WAHS-fearful (*n* = 192)**
Age	33.10 (12.07)	0.092	0.009
**Gender:**			
Female, other	237(60%)	Ref	Ref
Declined to answer	3(1%)	Excluded	Excluded
Male	158(40%)	−0.048	−0.094
**Race/ethnicity:**			
Hispanic	12(3%)	Ref	Ref
Asian	10(3%)	Ref	Ref
Black	12(3%)	Ref	Ref
Multiracial	15(4%)	Ref	Ref
Declined to answer	1 (< 1%)	Excluded	Excluded
White, no other race	348(87%)	−0.060	0.081
**Education:**			
High school diploma, GED or lower	88(22%)	Ref	Ref
Some college or associate’s degree	141(35%)	0.033	−0.198^∗^
Bachelor’s degree	116(29%)	0.039	−0.224^∗∗^
Graduate degree	53(13%)	0.001	−0.090
**Marital Status:**			
Declined to answer	2(1%)	Excluded	Excluded
Married	94(24%)	Ref	Ref
Long-term relationship	125(31%)	0.118	0.059
Single	177(44%)	0.118	−0.039
**Region:**			
United States	70(18%)	Ref	Ref
Europe	267(67%)	−0.126	−0.184^∗^
Other region or country	61(15%)	−0.042	−0.006
No Cost-related Non-adherence to Medical Care	198(50%)	Ref	Ref
Cost-related Non-adherence to Medical Care	199(50%)	0.449^∗∗^	0.414^∗∗^
Missing non-adherence questions	1 (< 1%)	Excluded	Excluded
		*R*^2^ = 0.248	*R*^2^ = 0.256

*Single includes divorced, separated, never married and widowed. Ref, reference group, WAHS, worry about affording healthcare scale. ^∗^*p* < 0.05, ^∗∗^*p* < 0.01.*

### Psychometric Results

**FIGURE 1:**
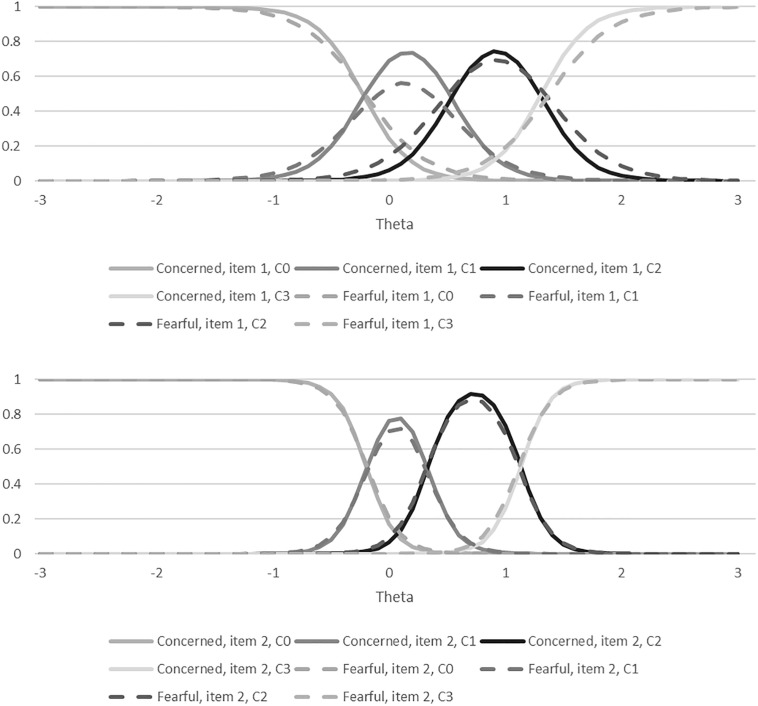
Item characteristic curves for the item response theory analyses. C, Category.

**FIGURE 2:**
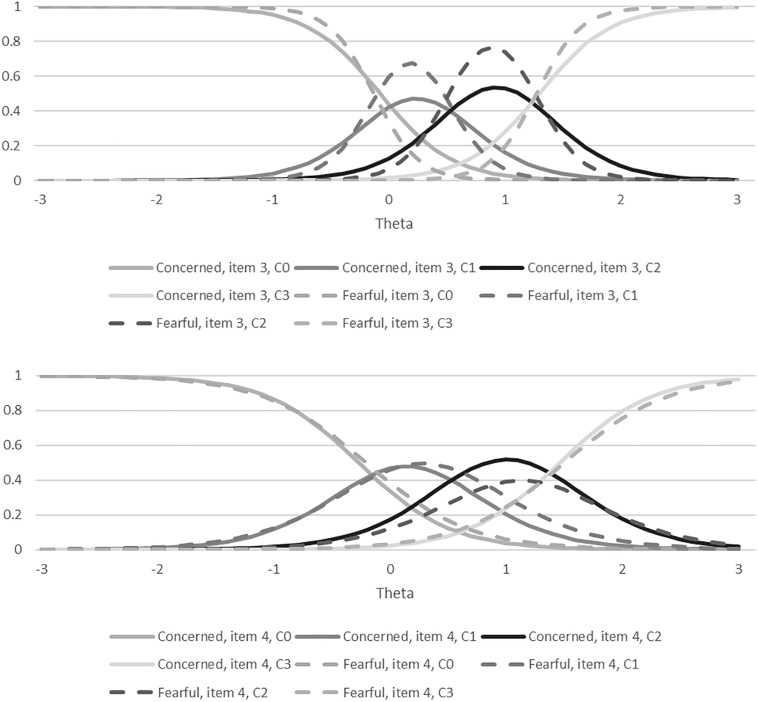
Information for the item response theory analyses.

**FIGURE 3:**
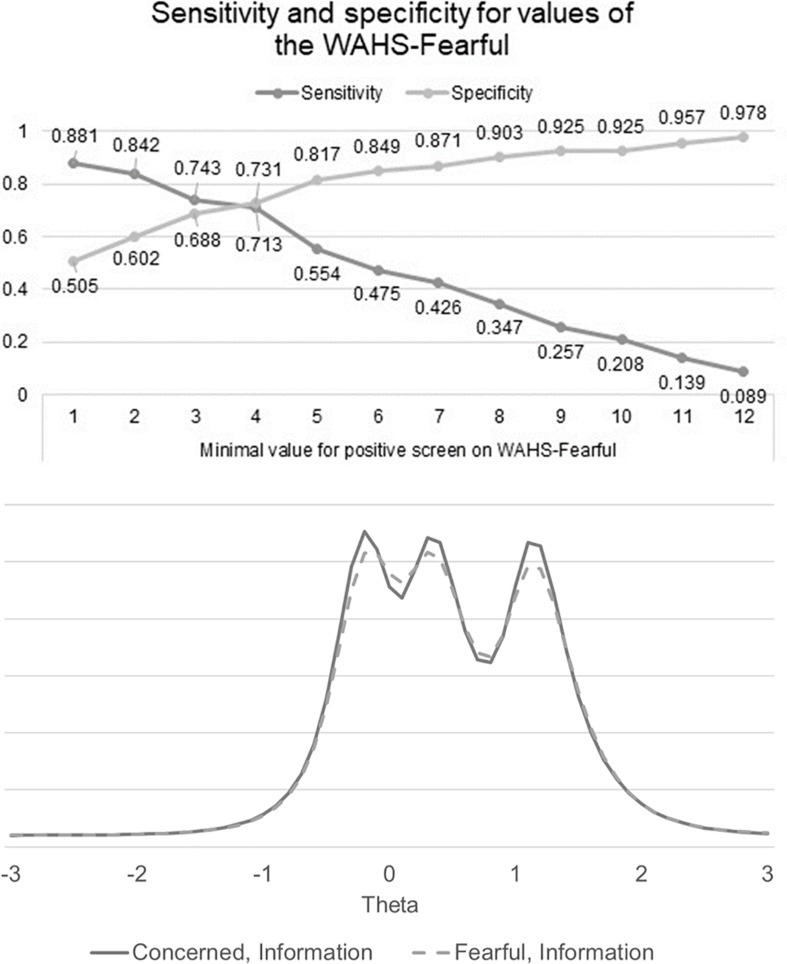
Sensitivity, specificity and information for values of the WAHS-fearful. The criterion was any cost-related non-adherence to medical care. WAHS, worry about affording healthcare scale.

The reliability and validity for the different versions of the WAHS are reported in Table 2. Cronbach’s alpha was over 0.9 for both versions, and both versions were significantly correlated with FAS, FWS, PROMIS-Anxiety Scale, the PROMIS QOL scale, and SCQ in the hypothesized directions. The DIF analyses did not show any statistically significant bias from the different wording on the WAHS items (all *p*’s > 0.05; see Figure 1 for item characteristic curves and Figure 2 for the information curve). The DIF IRT model had a root mean square error of approximation (RMSEA, an indicator of model fit to the data) of 0.04, indicating a good fit for the model (RMSEA’s <0.1) (Browne and Cudeck, 1993). Results for the multiple linear regression are reported in Table 1. In multivariate analyses, the WAHS-concerned was only significantly associated with cost-related non-adherence. In contrast, the WAHS-fearful was significantly associated with cost-related non-adherence, region and education level. The coefficients for cost-related non-adherence were large (WAHS-concerned: 0.449; WAHS-Fearful: 0414). The statistically significant coefficients for region and education with the WAHS-Fearful were small (−0.184 to −0.224). The means for the European participants were still 3.48 on a 0–12 scale, compared to 5.29 for American participants, indicating that despite greater likelihood of nationalized healthcare coverage, participants in Europe reported some minimal worry about affording healthcare. Because the WAHS-fearful showed better validity than the WAHS-concerned, we only conducted the ROC analysis on the WAHS-fearful. The area under the curve was 0.766 (95% confidence interval: 0.698 and 0.834). A score of four or greater on the WAHS-fearful had the best balance of sensitivity and specificity for detecting cost-related non-adherence (see Figure 3).

**TABLE 2:** Means, reliability, and validity for the worry about affording healthcare scale.

	**Concerned**	**Fearful**
Cronbach’s alpha	0.914	0.927
Spearman correlations		
General financial anxiety	–0.468^∗∗^	–0.470^∗∗^
Financial well-being scale	–0.428^∗∗^	–0.393^∗∗^
General anxiety	0.336^∗∗^	0.267^∗∗^
Quality of life: Mental health	−0.158^∗^	−0.170^∗^
Medical comorbidities	0.272^∗∗^	0.221^∗∗^
Total sample mean (SD)	4.05 (3.74)	4.04 (3.92)
United States Mean (SD)	5.29 (3.70)	5.53 (3.76)
Europe mean (SD)	3.48 (3.58)	3.48 (3.84)
Other regions mean (SD)	4.91 (4.00)	5.04 (3.98)

*SD, Standard deviation. ^∗^*p* < 0.05, ^∗∗^*p* < 0.01.*

## Discussion

This study reported on the adaptation and validation of the WAHS for use in the general population. Two different versions of the wording were tested; using “fearful” was associated with more socioeconomic indicators than “concerned.” Overall, the scale was reliable and showed validity through associations with cost-related non-adherence, medical diagnoses and other financial burden measures. It is important to note that the relationship to cost-related non-adherence remained in the multivariate analysis that controlled for potential confounds. Because the word “fearful” was associated with more socioeconomic indicators, future studies should use this wording. As a score of four or higher provided the best balance of sensitivity and specificity for detecting cost-related non-adherence, clinical use of the WAHS should use this cutoff score.

The study had several limitations. The sample size was relatively small although most measure validation studies are of comparable size (Williams et al., 2004). Because an online survey platform was used, there was likely a selection bias for people who could access the internet and would be willing to participate on such a platform. However, online survey platforms can overcome other selection biases as these allow people to participate who are not able to travel to participate in research, have to move frequently or might not be able to complete a telephone survey for economic or health reasons. As we were unable to ask detailed questions about location to control for cost of living, we could not use income as a variable. Future studies should consider subjective income questions such as those on the European Social Survey (ESS Round 8: European Social Survey, 2018). The sample was also predominantly Caucasian and had to read English, limiting generalizability. The low level of worry about affording healthcare in this sample also means the WAHS may not have sufficient reliability for higher levels of worry. Although the less than ideal sensitivity and specificity were noted, the outcome (cost-related non-adherence) was not an exact measure of the screening measure (the WAHS) so perfect concordance would not be expected. The study was also cross-sectional and a longitudinal validation study of the measure is warranted.

The WAHS has several potential uses in future studies and clinical care. First, it could be used to examine the role of worry about affording healthcare in motivating or demotivating healthcare use, such as cancer screening. Worry about affording healthcare could also be investigated as a reason for purchasing or not purchasing health insurance. Although our results showed some initial differences between the United States and Europe, more detailed and rigorous comparisons are warranted. Participants in Europe still reported slight worry about affording healthcare suggesting this might be an area for future research. People in the United States may also have different levels of worry about affording healthcare depending on the region and health insurance coverage. Different disease groups can also be compared to each other as well as people without medical conditions. As the WAHS is a relatively short measure (four items), it could be added to national or international surveys without substantially increasing participant burden or used as a screening measure in clinical care. Overall, worry about affording healthcare is a fruitful area of focus for future research.

## Data Availability Statement

The datasets generated for this study are available on request to the corresponding author.

## Ethics Statement

All procedures performed in this study were in accordance with the ethical standards of the Institutional Research Committee (the Fred Hutchinson Cancer Research Center, Human Subjects Division, #8703) and with the 1964 Helsinki declaration and its later amendments or comparable ethical standards.

## Author Contributions

SJ: conceptualization of the study, data collection and analysis, drafted the manuscript, and interpretation of results. YD: conceptualization of analysis, drafted the manuscript, and interpretation of results. LP and NH: manuscript revisions and interpretation of results.

## Conflict of Interest

YD was employed by Bayer Healthcare U.S. LLC. The remaining authors declare that the research was conducted in the absence of any commercial or financial relationships that could be construed as a potential conflict of interest.
