# Supplementary material for: Assessing Worry About Affording Healthcare in a General Population Sample
Source: Front Psychol. 2019 Nov 22;10:2622. doi: 10.3389/fpsyg.2019.02622 (PMC6883904; doi:10.3389/fpsyg.2019.02622)
Supplement: Supplementary file 1 [file Table_1.DOCX]

Supplementary Material

How [concerned/fearful] are you RIGHT NOW, if at all, that...

|  | **Not at all [concerned/fearful]** | **Not too [concerned/fearful]** | **Somewhat [concerned/fearful]** | **Very [concerned/fearful]** |
| --- | --- | --- | --- | --- |
| **Your income will go down and you won’t be able to afford the cost of your current health services and medications** |  |  |  |  |
| **You won’t be able to afford the health care services you need** |  |  |  |  |
| **You won’t be able to afford the prescription drugs you need** |  |  |  |  |
| **You won’t be able to afford medical devices you need (such as wheelchairs or eyeglasses)** |  |  |  |  |

Inter-item correlations for WAHS-Concerned

|  | WAHS 1 | WAHS 2 | WAHS 3 | WAHS 4 |
| --- | --- | --- | --- | --- |
| WAHS 1 | 1.000 | .841 | .715 | .664 |
| WAHS 2 | .841 | 1.000 | .757 | .705 |
| WAHS 3 | .715 | .757 | 1.000 | .671 |
| WAHS 4 | .664 | .705 | .671 | 1.000 |

Inter-item correlations for WAHS-Fearful

|  | WAHS 1 | WAHS 2 | WAHS 3 | WAHS 4 |
| --- | --- | --- | --- | --- |
| WAHS 1 | 1.000 | .819 | .803 | .643 |
| WAHS 2 | .819 | 1.000 | .857 | .724 |
| WAHS 3 | .803 | .857 | 1.000 | .704 |
| WAHS 4 | .643 | .724 | .704 | 1.000 |
